# Genetic Variation and Gene Expression of the Antimicrobial Peptide Macins in Asian Buffalo Leech (*Hirudinaria manillensis*)

**DOI:** 10.3390/biology14050517

**Published:** 2025-05-08

**Authors:** Yunfei Yu, Lizhou Tang, Mingkang Xiao, Jingjing Yin, Tianyu Ye, Rujiao Sun, Rui Ai, Fang Zhao, Zuhao Huang, Gonghua Lin

**Affiliations:** 1 School of Life Sciences, Jinggangshan University, Ji’an 343009, China; yuyunf2023@163.com (Y.Y.); xmk18379773872@163.com (M.X.); m15656142610@163.com (J.Y.); yetianyu0220@163.com (T.Y.); sunrujiao2022@163.com (R.S.); 18008109213@163.com (R.A.); zf_lgh@163.com (F.Z.); 2 School of Life Sciences, Jiangxi Normal University, Nanchang 330022, China; tanglizhou@163.com

**Keywords:** *Hirudinaria manillensis*, leech, macin, antimicrobial peptide, genetic variation, gene expression

## Abstract

With the growing severity of antibiotic resistance, antimicrobial peptides demonstrate significant potential for medical applications. Here, we performed genome and transcriptome sequencing of 30 Asian buffalo leech (*Hirudinaria manillensis*) individuals and integrated data from three other leech species (*Whitmania pigra*, *Hirudo nipponia*, and *Hirudo medicinalis*) to investigate genetic variation and gene expression of *H. manillensis* macins. Three macins (Hman1, Hman2, and Hman3), along with their encoding genes (*Hman1*, *Hman2*, and *Hman3*), were identified in *H. manillensis*. Hman1 exhibited the highest similarity (63.5 ± 12.0%) to macins from other leeches, followed by Hman2 (57.8 ± 7.4%) and Hman3 (30.0 ± 3.5%). Both amino acid and codon sequences of Hman1 were conserved within the species, whereas Hman2 and Hman3 exhibited markedly higher variability. All *Hman1* sequences were translatable, while four *Hman2* and 28 *Hman3* sequences had degenerated into pseudogenes. Transcripts per million (TPM) values for *Hman1*, *Hman2*, and *Hman3* were 2196.63, 242.35, and 1.22, respectively. Total macin expression in *H. manillensis* was less than 1/20 of that in *W. pigra*. Based on sequence variation and expression patterns, we propose that Hman1 retains functionality while Hman2 and Hman3 have lost or are losing their antibacterial functions.

## Simple Summary

Antimicrobial peptides exhibit potent antimicrobial activity and low potential for inducing resistance, making them promising therapeutic candidates. Here, we performed genome and transcriptome sequencing of 30 *Hirudinaria manillensis* individuals and integrated analogous data of three other leech species (*Whitmania pigra*, *Hirudo nipponia*, and *Hirudo medicinalis*) to investigate genetic variation and gene expression of antimicrobial peptide macins in *H. manillensis*. Three macins (Hman1, Hman2, and Hman3) and their encoding genes (*Hman1*, *Hman2*, and *Hman3*) were identified. Hman1, but not Hman2 or Hman3, showed close phylogenetic relationships with macins from other leeches. The sequences of Hman1 were conserved among different individuals, whereas Hman2 and Hman3 exhibited significantly higher variability. All *Hman1* sequences were translatable, while several *Hman2* and most *Hman3* sequences were pseudogenized. The total macin gene expression in *H. manillensis* was less than 1/20 of that in *W. pigra*. *Hman1* demonstrated universal expression across individuals, *Hman2* exhibited restricted occurrence, while *Hman3* was undetectable in nearly all individuals. Based on sequence variation and expression patterns, we hypothesize that Hman1 retains functionality, while Hman2 and Hman3 have lost or are losing antibacterial functions.

## 1. Introduction

Leeches are collective terms for animals in the phylum Annelida, class Clitellata, and subclass Hirudinea [1]. Approximately 700 leech species occur globally, distributed across all continents except Antarctica. Most inhabit freshwater ecosystems, while a few occupy terrestrial grasslands or marine environments [2]. Medicinal leeches, a subgroup of sanguivorous species, have been utilized since ancient Egypt, as evidenced by their depictions in pharaonic tomb frescoes [3]. In Europe, *Hirudo medicinalis* Linnaeus, 1758 is the primary species employed in therapy. Treatment typically involves applying live *H. medicinalis* to targeted areas to relieve blood congestion and improve circulation via blood extraction [4]. In China, medicinal species, such as *Hirudo nipponia* Whitman, 1886 and *Whitmania pigra* Whitman, 1884, are used to treat blood-stasis amenorrhea, traumatic injuries, and arthritis. Modern studies have isolated numerous bioactive substances from leeches with anticoagulant, anti-inflammatory, and antibacterial properties [5,6,7]. Notably, hirudin, a peptide firstly isolated from *H. medicinalis*, is the most potent known thrombin inhibitor, exhibiting a significantly lower bleeding risk than heparin [5,8].

Animal digestive tracts host highly complex microbial communities. These microbes interact with the host and mutually influence each other. Hosts provide habitat and essential nutrients for the gut microbiota, which in turn aid food digestion, supply nutrients unavailable through host synthesis, and inhibit pathogenic bacteria. Early culture-based studies indicated that leech gut microbiomes are dominated by a single *Aeromonas* genus [9,10]. Molecular biology techniques have since advanced leech gut microbiology research. Paul et al. analyzed the *Hirudo verbena* Carena, 1820 gut microbiota via terminal restriction fragment length polymorphism, revealing *Aeromonas veronii* and a novel *Rikenellaceae* clone PW3 as dominant in both the crop and gut [11]. Michele et al. employed high-throughput metagenomic sequencing to confirm *Proteus* strains in leech digestive tracts [12]. Leech gut bacteria secrete digestive proteases, assist erythrocyte lysis, and combat pathogens [13]. However, when utilized as medical devices, leeches may harbor gut microorganisms capable of causing infections [14,15]. In 2022, Christoph documented the first fatal *Aeromonas aeruginosa* septicemia case following live leech therapy [16]. Consequently, prophylactic antibiotics are administered during treatments, though emerging resistance in leech-borne pathogens has reduced therapeutic efficacy [17,18,19].

The increasing antibiotic resistance of pathogenic bacteria necessitates novel antimicrobial development [20]. Antimicrobial peptides (AMPs), also termed host defense peptides, are components of innate immunity [21]. These small peptides exhibit antibacterial, antifungal, and antiviral functions [22], primarily by disrupting pathogenic bacterial cell walls/membranes to exert lethal effects. AMPs demonstrate low propensity for resistance induction and hold potential as next-generation antimicrobial agents [23,24]. However, cytotoxicity and hemolytic effects frequently impede their clinical translation. Leeches have emerged as a promising source of novel AMPs. Rapid AMP-discovery strategies now employ genomic/proteomic mining across organisms [25]. Leech-secreted antimicrobial peptides prevent blood spoilage during storage, implying low toxicity to blood cells. For instance, Lu et al. isolated RK22, an anti-*Staphylococcus aureus* AMP with minimal hemolytic activity, from *H. manillensis* [26]. Grafskaia et al. computationally annotated the *H. medicinalis* genome, identifying eight AMPs with antimicrobial efficacy, low cytotoxicity, and negligible hemolysis [27].

Macins are frequently distributed antimicrobial peptides in leeches. In 2004, Tasiemski et al. first identified a duck leech (*Theromyzon tessulatum* Müller, 1774) macin peptide named theromacin, which exhibited activity against Gram-positive bacteria [28]. In 2008, Schikorski et al. identified another active macin called neuromacin from *H. medicinalis* and confirmed its antimicrobial activity [7]. Later, Ding et al. also identified an active macin called hirudomacin from *H. nipponia* [29]. Interestingly, another study also identified a functional macin analogue (hydramacin-1) in a *Hydra* sp. [30], which is phylogenetically distant from leeches. The ubiquitous distribution of functional macin homologs in leech species, as well as other invertebrate species, indicates that this antimicrobial peptide may play an important role in the host’s innate immune defense in these animals.

The Asian buffalo leech (*Hirudinaria manillensis* Lesson, 1842) is widely distributed across the Philippines, Vietnam, Malaysia, and a few Chinese provinces such as Guangxi, Fujian, and Hunan [31]. This leech preferentially feeds on mammalian blood (including humans), surviving nearly a year post-feeding without additional feeding. *H. manillensis* retains ingested blood in its crop for prolonged periods and may secrete antimicrobials, such as RK22 [26], to prevent spoilage. Compared to other medicinal leeches, it demonstrates superior anticoagulant activity, with multiple antithrombotic substances identified. Moreover, *H. manillensis* possesses a significantly larger body size and faster growth rate than typical medicinal leeches [32]. Therefore, this species represents substantial potential for pharmaceutical development. Despite its recognized antithrombotic values [31,32], systematic investigations of antimicrobial peptides in *H. manillensis* have been relatively scarce. Here, we conducted genome and transcriptome sequencing of 30 individuals of *H. manillensis* and integrated analogous data of three other leech species (*H. medicinalis*, *H. nipponia*, and *W. pigra*). We investigated the genetic variation and gene expression of antimicrobial peptide macins of *H. manillensis*. This will provide a scientific basis for the development and exploitation of leech antimicrobial peptide resources.

## 2. Materials and Methods

### 2.1. Genome Sequencing and Macin Identification

Three populations of *H. manillensis* were sampled from Rongxian, Guangxi (E 110.57°, N 22.81°), Ding’an, Hainan (E 110.35°, N 19.53°), and Maoming, Guangdong (E 110.39°, N 21.83°). As a comparison, three populations of *W. pigra* were collected from Wuhan, Hubei (E 114.57°, N 30.59°), Ji’an, Jiangxi (E 115.34°, N 27.51°), and Baodi, Tianjin (E 117.48°, N 39.47°). Ten live leeches were randomly selected from each population, and the anterior part of each specimen was sectioned and total DNA was extracted using the DNeasy Blood and Tissue kit (QIAGEN, Hilden, Germany). Meanwhile, total RNA from each anterior part was extracted using the TRIzol RNA extraction kit (Thermo Fisher Scientific Inc., Waltham, MA, USA), followed by purification with the RNeasy Mini Kit (Qiagen, Chatsworth, CA, USA). The DNA and RNA libraries of approximately 350 bp in length were prepared using Illumina’s proprietary reagents and were resequenced using the Illumina NovaSeq 6000 sequencing platform (Illumina Inc., San Diego, CA, USA) with both directions of 150 bp reads.

Raw read quality control was conducted with fastp v0.20.0 [33] employing default parameters. Cleaned reads were subsequently assembled via MEGAHIT v1.2.9 [34], yielding genomic contigs for individual samples. We also used our previously published reference genomes of *H. manillensis* [32], *W. pigra* [35], and *H. nipponia* [36], together with the reference genome of *H. medicinalis* (GenBank accession number GCA_011800805.1), for the identification and extraction of macins. Query sequences comprised published macin homologs: theromacin (AAR12065.1) from *T*. *tessulatum*, neuromacin (A8V0B3.1) from *H. medicinalis*, and hirudomacin (QBK51064.1) from *H. nipponia*. Homology searches were performed using BLAST v2.13.0+ (TBLASTN) [37] against both newly assembled (*H. manillensis* and *W. pigra*) and published reference genomes (*H. manillensis*, *W. pigra*, *H. nipponia*, and *H. medicinalis*).

### 2.2. Interspecific Variation Analysis

Macin coding sequences (CDSs) and their corresponding amino acid sequences (AASs) derived from the published genome of *H. manillensis*, *W. pigra*, *H. nipponia* [32,35,36], and *H. medicinalis* (GenBank accession number GCA_011800805.1) were aligned using MEGA v11.0.13 [38]. Interspecific pairwise similarity percentages of CDSs and AASs were calculated separately using Clustal Omega software v1.2.4 [39]. The subcellular localization of the macins was predicted using the online tool CELLO v2.5 (http://cello.life.nctu.edu.tw/, accessed on 20 March 2025) [40]. Phylogenetic trees were constructed based on maximum likelihood using IQ-TREE v1.6.12 [41], and the confidence level of each node was tested with 1000 rapid bootstrap tests. Amino acid and nucleotide substitution model tests were performed separately using the ModelFinder program integrated within IQ-TREE software v1.6.12 to select the optimal model [41]. The signal peptide region of each macin was identified using SignalP v6.0 [42] and subsequently removed using MEGA. The retained functional domains were then analyzed for the number of cysteines and glycines, the isoelectric point (pI), instability index (II), and grand average of hydropathicity (GRAVY) using the online tool ProtParam (https://web.expasy.org/protparam, accessed on 22 March 2025).

### 2.3. Intraspecific Variation Analysis

Intraspecific variation analyses were conducted for macins of *H. manillensis* and *W. pigra* by integrating CDSs and AASs from 30 individuals per species. DNA mutation sites and codon mutation sites were directly counted using MEGA. The frameshift mutations and nonsense mutations, which resulted in translational errors, were totaled as “malfunctional codons”. Approximately 100 bp of sequence was cut upstream or downstream of each variant site, and the matching reads were extracted from the original clean reads file using Mirabait v4.9.6 [43]. The gep function in SeqKit v0.10.2 [44] was used to query the matching sequence information to determine if each variant site was authentic. The nucleotide diversity index for each gene was computed with DnaSP v6.12.03 [45]. Haplotype relationships for each gene were plotted using PopART v1.7 [46].

### 2.4. Gene Expression Analysis

A comparative analysis of macin gene expression levels between *H. manillensis* and *W. pigra* was performed using transcriptomic data sequenced in this study. Reference sequences comprised CDSs from all predicted genes, including novelly identified macin genes and previously annotated genes derived from whole-genome structural annotations [32,35]. Transcripts per million (TPM) values were calculated for each gene using Salmon v1.0.0 [47], serving as quantitative measures of gene expression levels. Expression differences in between different macin genes were compared using SPSS v25.0 (IBM Corp., Armonk, NY, USA). Non-parametric methods were used to test the significance of differences. The Multiple Related Samples Test (Friedman test) was initially applied to assess overall inter-gene expression variation within each species. If the overall difference was significant (*p* < 0.05), the pairwise differences of each of two genes were further compared using the Two-Related-Samples method (Wilcoxon test). Furthermore, aggregated TPM values of all macin genes per individual were subjected to an interspecific comparison via the Two-Independent-Samples Test (Mann–Whitney U test).

## 3. Results

### 3.1. Basic Sequence Information

Three macins, designated Hman1, Hman2, and Hman3, with lengths of 83, 84, and 81 codons, including the stop codon, were identified in the *H. manillensis* reference genome. According to the GenBank online BLASTP search, Hman1 and Hman3 exhibited the highest sequence similarity to neuromacin (A8V0B3.1) from *H. medicinalis*, while Hman2 showed the highest similarity to hirudomacin (QBK51064.1) from *H. nipponia*.

Four macins were identified in the *H. nipponia* (Hnip1–Hnip4) and *H. medicinalis* (Hmed1–Hmed4) reference genomes, while five were identified in the *W. pigra* reference genome (Wpig1–Wpig5). All of these macins showed over 50% similarities to neuromacin from *H. medicinalis* or hirudomacin from *H. nipponia* (Table 1). The genes coding for all the above macin peptides had only one exon. The start codon of all sequences was ATG, and the stop codon of all sequences except Wpig5 (TAG) was TGA (Supplementary File S1).

### 3.2. Interspecific Variation

At the AAS level, Hman1 exhibited a similarity (mean ± standard deviation) of 63.5 ± 12.0 with the remaining 15 macin sequences; Hman2 demonstrated 57.8 ± 7.4, whereas Hman3 displayed substantially lower similarity at 30.0 ± 3.5. At the CDS level, *Hman1* showed 67.0 ± 9.8 similarity with other sequences, *Hman2* presented 61.7 ± 5.5, and *Hman3* maintained 44.3 ± 3.3. The comprehensive similarity matrices across all macin sequences are presented in Table 2.

**Figure 1:**
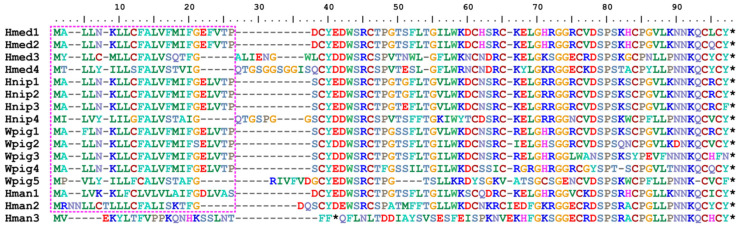
Alignment of amino acid sequences from *Hirudinaria manillensis* (Hman1–Hman3), *Whitmania pigra* (Wpig1–Wpig5), *Hirudo nipponia* (Hnip1–Hnip4), and *Hirudo medicinalis* (Hmed1–Hmed4). The purple frame indicates the signal peptide region; the black stars mean stop codons.

With the exception of *Hman3*, all *macin*s were successfully translated into intact AASs containing signal peptide regions. In contrast, Hman3 exhibited premature termination and had no detectable signal peptide region (Figure 1). Excluding Hman3, Wpig3, and Wpig5, most macin functional regions contained eight cysteine residues. The glycine count in each macin’s functional region ranges from 4 to 8, predominantly 6 (Table 3). The subcellular localization analysis showed that Hman3 was distributed in the nucleus, while other macins were localized extracellularly. All macins except for Wpig2 had isoelectric points above 7. All peptides were hydrophilic (with negative GRAVY values). Four peptides (Hman2, Hman3, Hmed4, Hnip4) showed instability indices exceeding 70 (Table 3).

**Figure 2:**
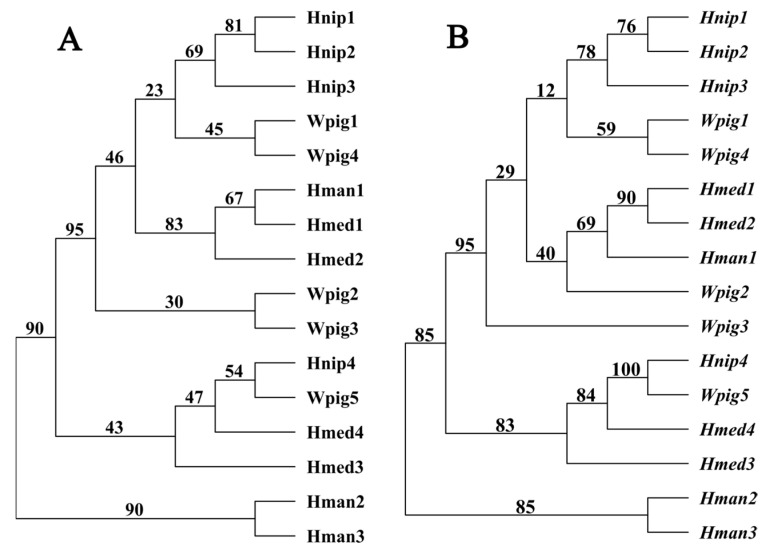
Phylogenetic relationships of different macins. (**A**) Based on amino acid sequence; (**B**) based on coding sequence. The numbers beside each node in the tree represent bootstrap percentages calculated through maximum likelihood analysis.

Phylogenetic analyses based on AASs revealed that Hman1 clustered in a monophyletic subclade containing all macins from the other three leech species, whereas Hman2 and Hman3 clustered in another monophyletic subclade (Figure 2A). The phylogenetic structure based on the CDSs was similar to that of AASs. The position of all genes except for *Hman1* and *Wpig2* on the phylogenetic tree remained almost unchanged (Figure 2B).

### 3.3. Intraspecific Variation

**Figure 3:**
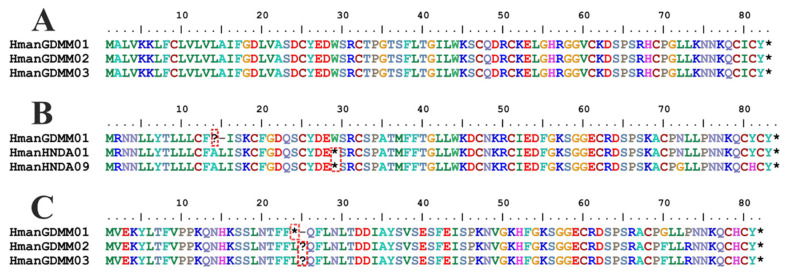
Examples of amino acid sequence variations in *H. manillensis* macins. (**A**) Hman1; (**B**) Hman2; (**C**) Hman3. The red frame indicates the malfunctional codons; the question marks show frameshift mutations sites; the black stars mean stop codons.

Complete CDSs of the Hman1, Hman2, and Hman3 peptides were obtained in all 30 *H. manillensis* samples (Supplementary File S2). *Hman1* exhibited relatively high conservation with only five variable sites, including one nonsynonymous codon, while the numbers of variable sites and nonsynonymous codons in *Hman2* and *Hman3* were much larger than those in *Hman1* (Table 4). All *Hman1* CDSs were translatable; interestingly, however, there were two and four malfunctional codons (premature termination or frameshift mutations), respectively, in *Hman2* and *Hman3* (Table 4), making several *Hman2* and most *Hman3* sequences degenerated into pseudogenes (Figure 3 and Supplementary File S2). Nucleotide diversity values for *Hman2* and *Hman3* were markedly elevated relative to *Hman1* (Table 4).

As a comparison, the CDSs of Wpig1–Wpig5 were also identified in 30 *W. pigra* individuals. There were 1–9 variable sites in each of the five CDS alignments, comprising 0–7 nonsynonymous codons. All CDSs of the five *W. pigra* macins were translatable with no malfunctional codons (Table 4). Notably, an 18 bp insertion (ACCACCGCTGCAAGGAAA, comprising six codons) was observed in the *Wpig5* sequences of 11 *W. pigra* specimens (Supplementary File S3). Due to the sequence conservation of this insertion, it was considered a single variant site. Overall, the intraspecific variation in Wpig1–Wpig5 was comparable to Hman1 but significantly lower than that observed in Hman2 and Hman3.

**Figure 4:**
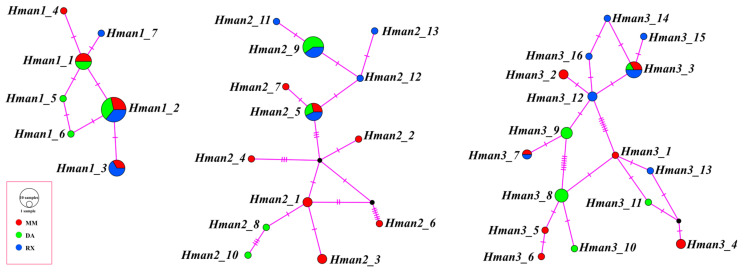
Haplotype network of the *H. manillensis* macin genes. The area of the circle represents the haplotype frequency; the small black circle indicates the median vector; the short purple horizontal line indicates the number of mutations.

There were seven haplotypes (*Hman1_1*–*Hman1_7*) of *Hman1* that formed a simple network. The maximum mutation number between neighboring haplotypes of *Hman1* was one. In contrast, *Hman2* and *Hman3* contained 13 (*Hman2_1*–*Hman2_13*) and 16 (*Hman3_1*–*Hman3_16*) haplotypes, respectively, with maximum mutation numbers between adjacent haplotypes being six and seven (Figure 4). The network structures of *Hman2* and *Hman3* were obviously more complex than that of *Hman1*. There were 2–13 haplotypes in *Wpig1*–*Wpig5*, with maximum mutation numbers between adjacent haplotypes being ≤ 3 (Supplementary Figure S1). The overall network architectures of *Hman2* and *Hman3* were markedly more complex than those of *Hman1* and *Wpig1*–*Wpig5*.

### 3.4. Gene Expression

Gene expression analysis based on the transcriptome data showed that *Hman1* had the highest mean expression level, followed by *Hman2* (Table 5). *Hman3* was barely expressed, having TPM values below 10 in all samples (Supplementary Table S1). The Friedman test indicated significant global expression variation among the three *H. manillensis* macins (χ^2^ = 58.207, *df* = 2, *p* < 0.001). A subsequent Wilcoxon Signed-Rank test confirmed that all pairwise expression differences between these genes were significant (*Z* ≤ −4.457, *p* < 0.05).

Among five *W. pigra* macin genes, *Wpig2* had the highest mean expression level, followed by *Wpig1*. *Wpig3* and *Wpig5* exhibited relatively low expression levels, whereas *Wpig4* was barely expressed (Table 5), with 29 out of 30 samples showing TPM values below 10 (Supplementary Table S2). The Friedman test revealed significant global expression variation among the five *W. pigra* macins (χ^2^ = 71.108, *df* = 4, *p* < 0.001). A subsequent Wilcoxon Signed-Rank test confirmed that most pairwise differences were significant (*Z* ≤ −2.201, *p* < 0.05), except for *Wpig1* vs. *Wpig2* (*Z* = −0.648, *p* = 0.517) and *Wpig3* vs. *Wpig5* (*Z* = −0.563, *p* = 0.573).

To compare total expression levels between *H. manillensis* and *W. pigra*, we aggregated TPM values of all macin genes of each individual. Results showed that the mean value of the total TPM of *W. pigra* macin genes was about 24-fold that of *H. manillensis* macin genes (Table 5). The Mann–Whitney U test confirmed that this interspecific difference was significant (*U* = 3.000, *p* < 0.001).

## 4. Discussion

In this study, we utilized genomic and transcriptomic data of *H. manillensis*, combined with analogous datasets of three additional leech species (*W. pigra*, *H. nipponia*, and *H. medicinalis*), to systematically investigate genetic variation and gene expression patterns of antimicrobial peptide macins of *H. manillensis*. Across these species, we identified 16 putative macin candidates. The NCBI online BLAST analysis revealed that all peptides exhibited >50% sequence similarity with either *H. medicinalis* neuromacin or *H. nipponia* hirudomacin, confirming their classification within the macin family. Notably, while previous studies on leeches and *Hydra* sp. reported only single macin members per species [7,28,29,30], our work identified 3–5 homologs in each examined species, demonstrating that macins constitute a multigene family. These findings suggest that macins are widely distributed across leech species and likely have more substantial antimicrobial functions in these organisms than previously recognized.

Generally, a higher number of multigene family members suggests greater functional importance of their peptide products [48]. Among the four leech species examined, *H. manillensis* exhibited the lowest macin gene count. Notably, several *Hman2* and most *Hman3* sequences were pseudogenized, suggesting the reduced antimicrobial functionality of macins in *H. manillensis* compared to other species. Sequence analyses revealed that Hman3 contained fewer cysteines and glycines, which are critical residues for maintaining protein stability and flexibility [49,50]. Subcellular localization analyses demonstrated Hman3’s nuclear retention, unlike other secreted macins. Moreover, Hman2 showed the highest instability index among all 16 macins. These findings collectively indicate the diminished functional significance of Hman2 and Hman3 compared to other macins.

Both sequence similarity and phylogenetic analyses showed that Hman1 and its encoding genes share closer evolutionary relationships with leech macins than Hman2/Hman3. Sequence similarity generally correlates with functional conservation, with >70% AAS identity conferring >90% probability of shared molecular functions [51,52]. Hman1 demonstrates 76.8% similarity to antimicrobial neuromacin (*H. medicinalis*), suggesting retained antibacterial activity. In contrast, Hman2/Hman3 show only ~67% similarity to active macins (neuromacin/hirudomacin) [7,29], combined with their frequent pseudogenization, strongly implying the potential loss of antibacterial functions.

Comparative genomic and transcriptomic analyses using *W. pigra* as a control revealed distinct patterns of intraspecific variation and expression profiles among *H. manillensis* macins. The results demonstrated that, similar to macins from *W. pigra*, both CDS and AAS sequences of Hman1 exhibited conservation within the species. In contrast, Hman2 and Hman3 displayed significantly higher intraspecific variability. Interestingly, all *Hman1* sequences maintained translatable integrity, whereas several *Hman2* and most *Hman3* sequences were identified as pseudogenes. We propose that relaxed selection pressure on Hman2 and Hman3 accounts for their elevated intraspecific variability. The total expression level of macin genes in *H. manillensis* was less than 1/20 of that observed in *W. pigra*. Furthermore, an expression analysis showed substantial disparities among macin genes in *H. manillensis*: *Hman1* contributed to approximately 90% of total expression, while *Hman2* and *Hman3* showed minimal or negligible expression. Based on sequence variation and expression patterns, we hypothesize that Hman1 retains functionality, whereas Hman2 and Hman3 have either lost or are progressively losing their antibacterial functions.

The leech microbiome represents a relatively specialized microbial community. Previous studies established that the *H. medicinalis* digestive tract microbiota primarily comprise *Aeromonas* and *Rikenella* [53,54]. *Aeromonas veronii* serves as the predominant pathogen in *W. pigra* [55], while the *H. nipponia* gastrointestinal microbiota are dominated by *Aeromonas*, *Streptococcus*, and *Mucinivorans* [29]. *Aeromonas*, a common opportunistic pathogen in aquatic organisms, can induce septicemia in immunocompromised hosts [56]. Although *Aeromonas* has been implicated in *H. manillensis* disease, its gastrointestinal colonization remains limited (<2%) [57], resulting in a reduced pathogenic threat compared to other leech species. Additionally, *H. manillensis* inhabits tropical low-latitude regions characterized by stable environmental parameters [58], which impose less physiological stress on aquatic species [59]. The combination of low *Aeromonas* abundance and stable tropical habitats likely diminishes the antimicrobial demands in this species. Given the metabolic cost of immune maintenance [60], we propose that the degeneration of Hman2 and Hman3 represents an adaptive response to tropical environments. Conversely, *W. pigra* occupies higher-latitude habitats with greater environmental fluctuations and faces heightened pathogenic pressure from *Aeromonas* [55], *Escherichia*, *Proteus*, and *Salmonella* [61]. These ecological factors necessitate both greater macin gene diversity and elevated expression levels in *W. pigra* compared to *H. manillensis*.

## 5. Conclusions

This study systematically investigated the genetic variation and gene expression of macins in *H. manillensis*. Three macins (Hman1–3) were identified. Hman1 exhibited phylogenetic conservation across leech species, low sequence variability, and robust expression, suggesting preserved antibacterial functionality. In contrast, Hman2 and Hman3 displayed high genetic divergence, pseudogenization, and limited expression, indicative of functional degeneration. We hypothesized that degeneration in Hman2 and Hman3 resulted from reduced pathogenic threats and physiological stress in the tropical habitats of *H. manillensis*. Our findings will help clarify the evolution of leech macins and provide critical insights for developing leech-derived antimicrobial peptides.

## Figures and Tables

**Table 1:** NCBI online BLAST results of the fifteen macins predicted in this study.

Species	Macin	Length	Target Species	Target Peptide	Accession	Identity *
*Hirudinaria manillensis*	Hman1	83	*H. medicinalis*	neuromacin	A8V0B3.1	76.8%
	Hman2	84	*H. nipponia*	hirudomacin	QBK51064.1	66.7%
	Hman3	81	*H. medicinalis*	neuromacin	A8V0B3.1	67.7%
*Hirudo medicinalis*	Hmed1	83	*H. medicinalis*	neuromacin	A8V0B3.1	98.8%
	Hmed2	83	*H. medicinalis*	neuromacin	A8V0B3.1	98.8%
	Hmed3	84	*H. nipponia*	hirudomacin	QBK51064.1	63.1%
	Hmed4	87	*H. nipponia*	hirudomacin	QBK51064.1	54.6%
*Hirudo nipponia*	Hnip1	83	*H. nipponia*	hirudomacin	QBK51064.1	95.1%
	Hnip2	83	*H. nipponia*	hirudomacin	QBK51064.1	95.1%
	Hnip3	83	*H. nipponia*	hirudomacin	QBK51064.1	93.9%
	Hnip4	85	*H. nipponia*	hirudomacin	QBK51064.1	61.9%
*Whitmania pigra*	Wpig1	83	*H. nipponia*	hirudomacin	QBK51064.1	90.2%
	Wpig2	83	*H. medicinalis*	neuromacin	A8V0B3.1	86.6%
	Wpig3	83	*H. medicinalis*	neuromacin	A8V0B3.1	82.5%
	Wpig4	82	*H. nipponia*	hirudomacin	QBK51064.1	80.5%
	Wpig5	79	*H. medicinalis*	neuromacin	A8V0B3.1	51.8%

* Sequence identity between each macin and its corresponding target peptide from BLAST analysis.

**Table 2:** Pairwise similarities of amino acid sequences and coding sequences of macins.

	Hman1	Hman2	Hman3	Hmed1	Hmed2	Hmed3	Hmed4	Hnip1	Hnip2	Hnip3	Hnip4	Wpig1	Wpig2	Wpig3	Wpig4	Wpig5
Hman1	—	59.0	32.1	77.1	77.1	60.2	56.6	72.3	72.3	71.1	56.6	71.1	69.9	63.9	64.6	48.1
Hman2	62.2	—	38.3	60.2	60.2	66.7	61.9	62.7	62.7	61.4	59.5	60.2	61.4	55.4	53.7	44.3
Hman3	49.0	51.4	—	29.6	29.6	34.6	32.1	29.6	29.6	28.4	29.6	29.6	29.6	22.2	27.2	27.8
Hmed1	77.9	61.8	43.6	—	98.8	62.7	57.8	90.4	90.4	89.2	62.7	89.2	86.7	79.5	80.5	54.4
Hmed2	76.3	61.4	42.8	98.0	—	62.7	57.8	90.4	90.4	89.2	62.7	89.2	86.7	79.5	81.7	54.4
Hmed3	61.4	68.3	47.7	60.2	60.6	—	72.6	65.1	65.1	63.9	61.9	61.4	62.7	57.8	53.7	50.6
Hmed4	60.6	64.3	45.7	61.4	61.4	77.4	—	59.0	59.0	59.0	70.6	55.4	55.4	51.8	50.0	53.2
Hnip1	74.3	65.1	44.9	89.2	88.4	62.7	64.7	—	100.0	97.6	62.7	94.0	86.7	80.7	80.5	53.2
Hnip2	74.7	65.5	44.4	89.6	88.8	62.2	64.3	99.6	—	97.6	62.7	94.0	86.7	80.7	80.5	53.2
Hnip3	73.9	64.7	43.6	89.6	88.8	61.8	63.9	98.4	98.8	—	61.4	92.8	85.5	80.7	79.3	54.4
Hnip4	57.8	57.5	39.1	64.3	63.9	60.7	69.0	63.9	64.3	63.9	—	59.0	59.0	54.2	50.0	57.0
Wpig1	72.3	65.1	44.9	89.2	88.4	60.6	62.7	93.6	94.0	94.0	63.9	—	86.7	81.9	84.1	53.2
Wpig2	73.5	64.7	43.2	89.2	88.0	61.4	62.2	92.8	92.4	92.4	62.2	91.6	—	79.5	78.0	51.9
Wpig3	72.7	64.7	43.2	85.5	85.5	61.8	63.5	91.2	90.8	90.8	61.0	89.6	90.4	—	75.6	45.6
Wpig4	70.3	61.0	41.6	85.4	85.0	57.3	59.3	88.6	89.0	89.0	58.9	89.4	87.8	87.0	—	48.1
Wpig5	47.7	48.1	39.2	57.0	57.0	54.4	62.4	57.8	58.2	58.2	71.7	58.2	56.1	54.0	54.4	—

Note: the dash (—) denotes omitted comparisons between identical sequences, the above diagonal indicates the pairwise similarity of amino acid sequences, and the below diagonal indicates pairwise similarity of coding sequences.

**Table 3:** Basic information of macin peptides.

Species	Macin	Cys	Gly	PSL	Reliability	pI	GRAVY	II
*H. manillensis*	Hman1	8	6	Extracellular	4.253	8.49	−0.705	59.53
	Hman2	8	5	Extracellular	4.309	7.68	−0.798	103.89
	Hman3	4	4	Nuclear	1.819	7.68	−0.619	73.42
*H. medicinalis*	Hmed1	8	6	Extracellular	3.968	8.49	−0.705	55.00
	Hmed2	8	6	Extracellular	4.100	8.49	−0.829	49.18
	Hmed3	8	6	Extracellular	4.304	7.71	−0.717	62.77
	Hmed4	8	9	Extracellular	4.522	8.18	−0.777	75.11
*H. nipponia*	Hnip1	8	7	Extracellular	4.249	9.06	−0.785	63.71
	Hnip2	8	7	Extracellular	4.249	9.06	−0.785	63.71
	Hnip3	8	6	Extracellular	4.220	8.90	−0.768	68.41
	Hnip4	8	7	Extracellular	4.226	8.49	−0.580	70.69
*W. pigra*	Wpig1	8	6	Extracellular	4.196	8.90	−0.781	60.14
	Wpig2	8	6	Extracellular	4.052	6.46	−0.451	64.15
	Wpig3	5	5	Extracellular	3.523	8.36	−0.869	62.77
	Wpig4	8	8	Extracellular	3.775	8.72	−0.472	57.44
	Wpig5	7	5	Extracellular	3.980	7.72	−0.212	36.85

Note: PSL, protein subcellular localization; pI, isoelectric point; GRAVY, grand average of hydropathicity; II, Instability index.

**Table 4:** Intraspecific genetic variations in macins of *H. manillensis* (Hman1–Hman3) and *W. pigra* (Wpig1–Wpig5).

Macin	Variable Sites	Nonsynonymous Codons	Malfunctional Codons	Nucleotide Diversity
Hman1	5	1	0	0.00413
Hman2	24	16	2	0.01722
Hman3	25	10	4	0.02309
Wpig1	1	0	0	0.00162
Wpig2	9	6	0	0.00992
Wpig3	9	7	0	0.01052
Wpig4	7	4	0	0.00446
Wpig5	6	4	0	0.00359

**Table 5:** The expression levels of the *macins*.

	*Macin*	TPM (Mean ± SD)	Total TPM (Mean ± SD)
*H. manillensis*	*Hman1*	2196.63 ± 1033.77 ^a^	2440.20 ± 1048.87
	*Hman2*	242.35 ± 371.24 ^b^	
	*Hman3*	1.22 ± 2.50 ^c^	
*W. pigra*	*Wpig1*	22,278.23 ± 18,671.18 ^A^	59,433.26 ± 82,551.14
	*Wpig2*	37,089.85 ± 85,607.08 ^A^	
	*Wpig3*	45.10 ± 195.60 ^B^	
	*Wpig4*	1.96 ± 4.09 ^C^	
	*Wpig5*	18.11 ± 40.69 ^B^	

Note: different superscript letters indicate significant within-group differences (Wilcoxon Signed-Rank test, *p* < 0.05).

## Data Availability

Data are contained within the article and Supplementary Files.

## Supplementary Materials

The following supporting information can be downloaded at: https://www.mdpi.com/article/10.3390/biology14050517/s1, File S1: Macin CDSs in reference genomes of four leech species; File S2: Macin CDSs obtained in 30 H. manillensis samples; File S3: Macin CDSs obtained in 30 W. pigra samples; Figure S1: Haplotype network of the W. pigra macin genes; Table S1: TPM values of macin genes of 30 H. manillensis samples; Table S2: TPM values of macin genes of 30 Whitmania pigra samples.

## Abbreviations

AMP	antimicrobial peptide
CDS	coding sequence
AAS	amino acid sequence
TPM	transcripts per million
PSL	protein subcellular localization;
pI	isoelectric point
GRAVY	grand average of hydropathicity
II	instability index
