# Supplementary figures and images for: Genetic Variation and Gene Expression of the Antimicrobial Peptide Macins in Asian Buffalo Leech (Hirudinaria manillensis)
Source: Biology (Basel). 2025 May 8;14(5):517. doi: 10.3390/biology14050517 (PMC12109416; doi:10.3390/biology14050517)

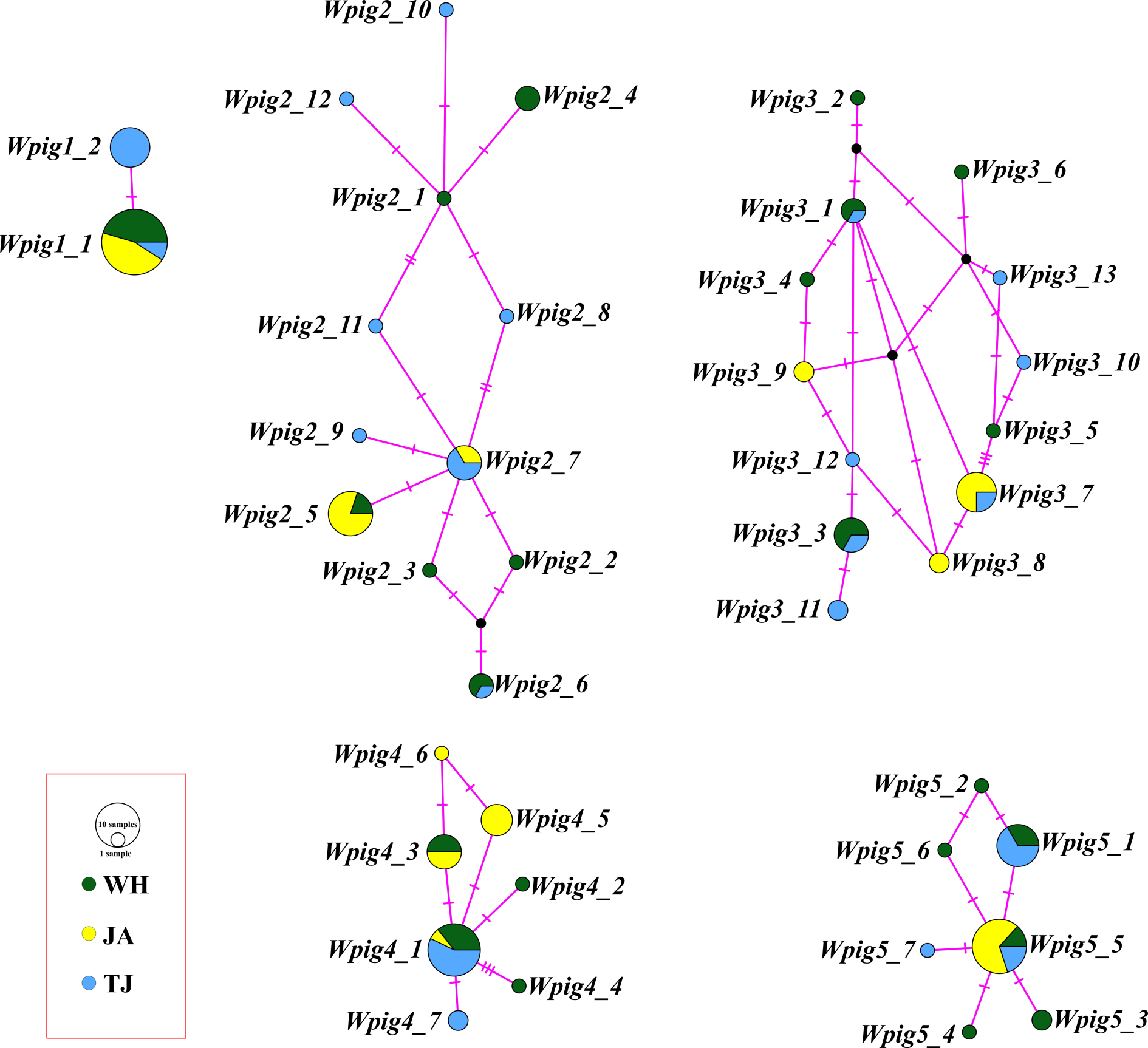

Supplement: Supplementary file 1 [file biology-14-00517-s001.zip › Figure S1.tiff]
